# Dynamic Self-Consistent Field Approach for Studying Kinetic Processes in Multiblock Copolymer Melts

**DOI:** 10.3390/polym12102205

**Published:** 2020-09-25

**Authors:** Friederike Schmid, Bing Li

**Affiliations:** Institut für Physik, Johannes Gutenberg-Universität Mainz, D 55099 Mainz, Germany; binli@uni-mainz.de

**Keywords:** dynamic density functional theory, single chain structure factor, multiblock copolymers, two-length scale copolymers, ordering kinetics

## Abstract

The self-consistent field theory is a popular and highly successful theoretical framework for studying equilibrium (co)polymer systems at the mesoscopic level. Dynamic density functionals allow one to use this framework for studying dynamical processes in the diffusive, non-inertial regime. The central quantity in these approaches is the mobility function, which describes the effect of chain connectivity on the nonlocal response of monomers to thermodynamic driving fields. In a recent study, one of us and coworkers have developed a method to systematically construct mobility functions from reference fine-grained simulations. Here we focus on melts of linear chains in the Rouse regime and show how the mobility functions can be calculated semi-analytically for multiblock copolymers with arbitrary sequences without resorting to simulations. In this context, an accurate approximate expression for the single-chain dynamic structure factor is derived. Several limiting regimes are discussed. Then we apply the resulting density functional theory to study ordering processes in a two-length scale block copolymer system after instantaneous quenches into the ordered phase. Different dynamical regimes in the ordering process are identified: at early times, the ordering on short scales dominates; at late times, the ordering on larger scales takes over. For large quench depths, the system does not necessarily relax into the true equilibrium state. Our density functional approach could be used for the computer-assisted design of quenching protocols in order to create novel nonequilibrium materials.

## 1. Introduction

Block copolymers, i.e., polymers made of different chemically incompatible units, are known to spontaneously self-assemble into a rich variety of nanostructured patterns [1,2,3]. The morphologies and dimensions of these morphologies can be varied by tuning the molecular weight and architecture of the constituent polymers. This makes them interesting for many applications such as drug delivery [4], energy conversion [5,6], or soft lithography [7,8], as well as for fundamental research.

Theoretically, the self-consistent field (SCF) theory has proved to be a particularly valuable tool for studying self-assembled structures and morphological phase diagrams [9,10,11,12]. In parameter regimes where thermal fluctuations can be neglected, SCF models can often predict equilibrium self-assembled structures at a quantitative level. However, real materials often do not reach the true, fully ordered equilibrium state on experimental time scales. Defects form during the ordering process, which do not annihilate unless special techniques are applied [13,14,15,16,17,18,19,20]. Furthermore, intermediate states may appear, which may be interesting by themselves and can stabilized by crosslinking or freezing. The properties of these transition states not only depend on the characteristics of the constituent molecules, but also on the way the material is processed. For these reasons, considerable effort is also spent on studying the dynamics of block copolymer (BCP) ordering processes [21].

SCF theories are often derived by field theoretical methods, i.e., first rewriting the partition function as a functional integral via insertion of Delta functionals, and then applying a saddle-point approximation. Similar approaches have recently been taken to derive a dynamic SCF theory [22,23], starting from the Martin–Siggia–Rose functional for Langevin dynamics [24]. Solving the resulting dynamic SCF equations typically involves simulating an ensemble of independent chains moving in a co-evolving self-consistent field [25], similar to the ‘self-consistent Brownian dynamics’ [26,27,28], ‘single-chain in mean field’ [29], or ‘MD-SCF’ simulation methods [30] that have been used with great success to study polymer systems in and out of equilibrium.

Another popular class of dynamic extensions of the SCF theory is the class of dynamic self-sonsistent field or dynamic density functional theories (DDFTs), which combine the free energy functional of the SCF theory with a diffusive dynamical model for the polymer relaxation and do not require explicit chain simulations. The generic form of dynamical equation of an inhomogeneous (co)polymer system has the form [31,32,33,34]

(1) $$\partial_{t}\underline{\rho }\left(r,t\right)=\nabla_{r}\int dr^{\prime }\;\underline{\underline{\Lambda }}\left(r,r^{\prime }\right)\nabla_{r^{\prime }}\underline{\mu }\left(r^{\prime },t\right)$$

where $\underline{\rho }\left(r,t\right)=\left(\rho_{\alpha }\left(r,t\right)\right)$ denotes the local densities at position r and time *t* of monomers of type α in vector notation, $\underline{\underline{\Lambda }}\left(r,r^{\prime }\right)=\left(\Lambda_{\alpha \beta }\left(r,r^{\prime }\right)\right)$ a mobility matrix, and $\underline{\mu }\left(r^{\prime }\right)=\left(\mu_{\beta }\left(r^{\prime }\right)\right)$ is derived from the SCF free energy functional $F\{\underline{\rho }\}$ via $\mu_{\beta }\left(r^{\prime },t\right)=\delta F/\delta \rho_{\beta }\left(r^{\prime },t\right)$. Hydrodynamics can be included by adding a convective term [35] to Equation (1) and combining it with a dynamical equation for fluid flow [36,37].

The mobility matrix relates the local thermodynamic force $\left(-\nabla_{r^{\prime }}\underline{\mu }\left(r^{\prime },t\right)\right)$ on monomers at position $r^{\prime }$ to the monomer density current at position r, taking into account the effect of chain connectivity. It thus incorporates the information on polymer dynamics, e.g., internal chain relaxation and possibly entanglements. It should be noted that an “exact” mobility matrix should also depend on frequency according to the Mori–Zwanzig theory [38,39,40]. A generalized dynamic RPA (random phase approximation) theory that includes memory has recently been proposed by Wang et al. [41]. The central assumption of Equation (1) is that one can describe inhomogeneous polymer systems by an effective Markovian model which accounts for the multitude of relaxation time scales in polymer systems in terms of a suitable (effective) nonlocal mobility matrix.

The question is how to determine this mobility matrix. A number of expressions have been proposed in the literature [31,32,34,42,43,44], which rely on more or less heuristic assumptions. On the other hand, it was found that not only the time scales, but also the pathways of self-assembly may depend critically on the specific choice of the mobility matrix [45,46]. In a previous paper, we have therefore developed a more systematic approach, where the mobility matrix is constructed in a bottom-up manner from the single chain dynamic structure factor in particle-based reference simulations [47]. We have tested it at the example of diblock copolymer melts with lamellar ordering and shown that DDFT calculations based on our approach can accurately reproduce the ordering and disordering kinetics in these systems. In fact, the DDFT results and the corresponding computer simulation data were found to be in similar quantitative agreement than SCF predictions and computer simulation data for equilibrium structures.

In Ref. [47], the mobility matrix was determined from fine-grained simulation data. However, if reliable theoretical expressions for the single chain dynamic structure factor are available, our approach can also be used to derive analytic or semi-analytic expressions for the mobility matrix, without having to resort to fine-grained reference simulations. The purpose of the present paper is to provide such a description for melts of linear multiblock copolymers in the Rouse regime, i.e., the regime where chains are not entangled. We will first discuss the dynamic structure factor of Rouse copolymers and present a highly accurate analytical approximate expression, which can be used for efficiently calculating the mobility matrix of linear multiblock copolymers with arbitrary block sequence. To illustrate our approach, we will then apply the dynamic theory to a particularly interesting multiblock copolymer melt with two competing length scales [48,49], and show how the competition affects the pathways of self-assembly and the resulting final structures.

## 2. Theory

We consider melts of Gaussian chains of total length *N* in the Rouse regime at total monomer density $\rho_{0}$. Single non-interacting chains are characterized by their radius of gyration $R_{g}$ and the chain diffusion constant $D_{c}$, or, alternatively, the Rouse time $\tau_{R}=\frac{2}{\pi^{2}}R_{g}^{2}D_{c}$. Monomers have different types α, and the monomer sequence along the chains is described by a function $\underline{\chi }\left(n/N\right)$, with $\chi_{\alpha }\left(n/N\right)=1$ if monomer *n* is of type α, and $\chi_{\alpha }\left(n/N\right)=0$ otherwise ($\sum_{\alpha }\chi_{\alpha }\left(n/N\right)\equiv 1$). Knowing $\underline{\chi }$, one can calculate the overall fraction of monomers α in the chain $f_{\alpha }=\int_{0}^{1}d\tilde{n}\;\chi_{\alpha }\left(\tilde{n}\right)$. The free energy of the melt is described by a free energy functional $F\{\phi_{\alpha }\left(r,t\right)\}$, which depends on the rescaled local densities of type α monomers, $\phi_{\alpha }\left(r,t\right)=\rho_{\alpha }\left(r,t\right)/\rho_{0}$. In practice, we will consider block copolymers made of two types of monomers A and B, with Edwards-type interactions characterized by a Flory–Huggins parameter χ and a Helfand compressibility parameter κ [50], and use the SCF free energy functional describing this class of systems. The relevant equations are summarized in Appendix A.

As in Ref. [47], we will use reduced quantities $\underline{\phi }=\underline{\rho }/\rho_{0}$, $\hat{\underline{\mu }}=N\underline{\mu }$ and $\hat{\underline{\underline{\Lambda }}}=\underline{\underline{\Lambda }}/\rho_{0}N$ to simplify the notation. Equation (1) then takes the form

(2) $$\partial_{t}\underline{\phi }\left(r,t\right)=\nabla_{r}\int dr^{\prime }\;\hat{\underline{\underline{\Lambda }}}\left(r,r^{\prime }\right)\nabla_{r^{\prime }}\hat{\underline{\mu }}\left(r^{\prime },t\right)$$

The thermodynamic driving field ${\hat{\mu }}_{\alpha }\left(r,t\right)=\frac{N}{\rho_{0}}\delta F/\delta \phi_{\alpha }\left(r,t\right)$ is derived from the SCF functional of the copolymer system. The corresponding equations are given in Appendix A.

Following Ref. [47], we approximate the mobility matrix by that of a homogeneous reference system. This implies, in particular, that it is translationally invariant, $\hat{\underline{\underline{\Lambda }}}\left(r-r^{\prime }\right)$, hence we can conveniently rewrite Equation (2) in Fourier space as

(3) $$\partial_{t}\underline{\phi }\left(q,t\right)=-q^{2}\;\hat{\underline{\underline{\Lambda }}}\left(q\right)\;\hat{\underline{\mu }}\left(q,t\right)$$

with the Fourier transform defined via $f\left(q\right)=\int dre^{iq\cdot r}f\left(r\right),\;f\left(r\right)=\frac{1}{V}\sum_{q}e^{-iq\cdot r}f\left(q\right)$

We determine $\hat{\underline{\underline{\Lambda }}}\left(q\right)$ using the ”relaxation time approach” developed in Ref. [47], i.e., we calculate it from the characteristic relaxation times of the single-chain dynamic structure factor $\underline{\underline{g}}\left(q,t\right)$ in the reference system:

(4) $$\hat{\underline{\underline{\Lambda }}}\left(q\right)=\frac{1}{k_{B}TN^{2}}\;\underline{\underline{g}}\left(q,0\right)\;{\underline{\underline{G}}}^{-1}\left(q\right)\;\underline{\underline{g}}\left(q,0\right)\;\mathrm{with}\;\underline{\underline{G}}\left(q\right)=\frac{q^{2}}{N}\int_{0}^{\infty }dt\;\underline{\underline{g}}\left(q,t\right)$$

This expression has been constructed such that the DDFT consistently reproduces $\underline{\underline{g}}\left(q,t\right)$ when used to study the relaxation dynamics of a single tagged chain. Further details can be found in Ref. [47].

**Figure A1:**
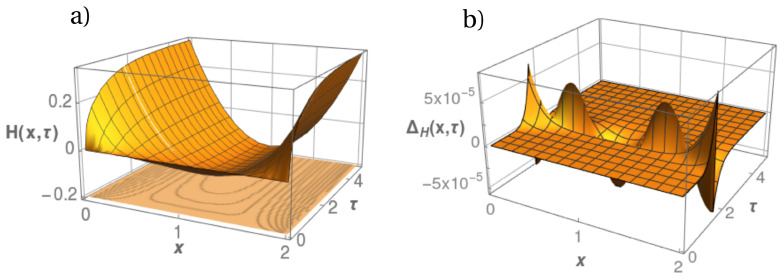
Illustration of (**a**) H(x,τ) and (**b**) deviation $\Delta \left(x,\tau \right)=H_{appr}\left(x,\tau \right)-H\left(x,\tau \right)$ between the approximate expression, Equation (A21) and the numerical value, Equation (A12), in the range x∈[0:2].

The central input quantity is thus the single chain dynamic structure, defined as

(5) $$\underline{\underline{g}}\left(q,t\right)=\frac{1}{N}\;\int \int_{0}^{N}\;\;\;dn\;dm\;\underline{\chi }\left(n/N\right)⊗\underline{\chi }\left(m/N\right)\langle e^{iq\cdot (R_{n}\left(t\right)-R_{m}\left(0\right))}\rangle$$

where 〈·〉 denotes the configurational average over all chain conformations, ⊗ the tensor product, and $R_{n}\left(t\right)$ gives the coordinates of monomer *n* at time *t*. In Ref. [47], we propose to measure $\underline{\underline{g}}\left(q,t\right)$ from reference particle-based simulations. Here, we take an alternative approach and estimate it from the analytical solution for free Gaussian Rouse chains. For homopolymers, an exact expression is available [51], which has been discussed extensively in the literature in various limiting regimes [41,51,52]. The generalization to block copolymers is straightforward (see Appendix B.2). However, using the resulting expression in the above formalism is not easy, because it involves an infinite sum over Rouse modes. To overcome this problem, we have derived an approximate expression, which avoids the sum, but still accurate reproduces $\underline{\underline{g}}\left(q,t\right)$ over the whole relevant range of q and *t*. The derivation can be found in the Appendix B.1. The result is

(6) $$\begin{array}{ccc} \underline{\underline{g}}\left(q,t\right) & = & \;N\;\;\int \int_{0}^{1}\;\;\;d\tilde{n}\;d\tilde{m}\;\underline{\chi }\left(\tilde{n}\right)⊗\underline{\chi }\left(\tilde{m}\right)\;{\tilde{g}}_{\tilde{n}\tilde{m}}\left(qR_{g},t/\tau_{R}\right)\\ \mathrm{with}\;{\tilde{g}}_{\tilde{n}\tilde{m}}\left(\tilde{q},\tilde{t}\right) & = & \left\{\begin{array}{cc} e^{-{\tilde{q}}^{2}\left|\tilde{n}-\tilde{m}\right|}\;e^{-{\tilde{q}}^{2}\sqrt{\tilde{t}}\left(\Phi \left(\frac{\tilde{n}-\tilde{m}}{\sqrt{\tilde{t}}}\right)+\Phi \left(\frac{\tilde{n}+\tilde{m}}{\sqrt{\tilde{t}}}\right)+\Phi \left(\frac{2-|\tilde{n}+\tilde{m}|}{\sqrt{\tilde{t}}}\right)+\Phi \left(\frac{2-\tilde{n}-\tilde{m}}{\sqrt{\tilde{t}}}\right)\right)} & :\tilde{t}<\tau^{*}\\ e^{-{\tilde{q}}^{2}W\left(\tilde{n}\right)}\;e^{-{\tilde{q}}^{2}W\left(\tilde{m}\right)}\;e^{-{\tilde{q}}^{2}\frac{2}{\pi^{2}}\left(\tilde{t}-2\cos\left(\pi \tilde{n}\right)\cos\left(\pi \tilde{m}\right)\;e^{-\tilde{t}}\right)} & :\tilde{t}>\tau^{*} \end{array}\right. \end{array}$$

(7) $$\mathrm{and}\;W\left(\tilde{n}\right)={\tilde{n}}^{2}-\tilde{n}+\frac{1}{3},\;\Phi \left(y\right)=\frac{2}{\pi^{2}}\;\left(e^{-\frac{1}{4}{\left(\pi y\right)}^{2}}\sqrt{\pi }-\frac{\pi^{2}}{2}|y|\;(1-\mathrm{Erf}(\pi |y|/2))\right)$$

where Erf is the error function, and the scaled crossover time is set to $\tau^{*}=1.7$. As demonstrated in Appendix B.2, the relative error of ${\tilde{g}}_{nm}$ with respect to the exact solution is less than $2{\left(qR_{g}\right)}^{2}\times 10^{-4}$ over the whole range of q and *t* (see Figure A1 in the Appendix B).

Equation (6) shows that the behavior of $\underline{\underline{g}}\left(q,t\right)$ features two time regimes: At small times $t<\tau^{*}$, the full spectrum of Rouse modes contributes to the dynamic structure factor in a collective manner that can be captured by a scaling function Φ(y). At large times $t>\tau^{*}$, only the leading Rouse mode contributes. In the limit t→∞, $\underline{\underline{g}}\left(q,t\right)$ assumes the asymptotic behavior

(8) $$\underline{\underline{g}}\left(q,t\right)\overset{t\to \infty }{⟶}N\;e^{-q^{2}D_{c}t}\;\underline{I}\left(qR_{g}\right)⊗\underline{I}\left(qR_{g}\right)\;\mathrm{with}\;\underline{I}\left(\tilde{q}\right)=\int_{0}^{1}\;\;\;d\tilde{n}\;\underline{\chi }\left(\tilde{n}\right)\;e^{-{\tilde{q}}^{2}\;W\left(\tilde{n}\right)}$$

This equation can also be derived independently, see Appendix B.1, Equation (A8). In the limit t→0 and $(qR_{g})\to \infty$, the double integral over $\tilde{n}$ and $\tilde{m}$ is dominated by the sharply peaked term $e^{-{\left(qR_{g}\right)}^{2}\left|\tilde{n}-\tilde{m}\right|}$, i.e., by contributions of monomers that are close along the chain, n≈m. In that limit, one obtains the scaling form

(9) gαβ(q,t)⟶t→0(qRg)→∞δαβ2fαN(qRg)2F(qRg)2t/τR

with the scaling function $F\left(x\right)=\int_{0}^{\infty }du\;e^{-u-x\Phi (u/x)}$, where Φ is defined as in Equation (7). This corresponds to Equation (4.III.12) in Ref. [51], generalized to linear multiblock copolymers.

Finally, at t=0, we have ${\tilde{g}}_{\tilde{n}\tilde{m}}=e^{-{\tilde{q}}^{2}\left|\tilde{n}-\tilde{m}\right|}$ for all $\tilde{q}$. For linear multiblocks containing a set $\left\{\alpha_{i}\right\}$ of blocks of type α with block length $Nb_{\alpha_{i}}$, the integral Equation (6) then gives

(10) gαα(q,0)=N(qRg)4{2∑αie−(qRg)2bαi−1+bαi(qRg)2+∑αi,αji≠je−bαi(qRg)2−1e−bαj(qRg)2−1e−Δαi,αj}

(11) $$\begin{array}{ccc} g_{\alpha \beta }\left(q,0\right) & = & \frac{N}{{\left(qR_{g}\right)}^{4}}\;\left\{\sum_{\alpha_{i},\beta_{j}}\left(e^{-b_{\alpha_{i}}{\left(qR_{g}\right)}^{2}}-1\right)\;\left(e^{-b_{\beta_{j}}{\left(qR_{g}\right)}^{2}}-1\right)\;e^{-\Delta_{\alpha_{i},\beta_{j}}}\right\} \end{array}$$

where $N\Delta_{\alpha_{i}\alpha_{j}}$ or $N\Delta_{\alpha_{i}\beta_{j}}$ is the number of segments separating the blocks ($\alpha_{i}$, $\alpha_{j}$) or ($\alpha_{i}$,$\beta_{j}$), respectively.

Using these results, we can now apply Equation (4) to evaluate the mobility function. In the regime $t/\tau_{R}>\tau^{*}$, the time integral can be evaluated analytically

(12) $${\tilde{q}}^{2}\;\int_{\tau^{*}}^{\infty }d\tilde{t}\;{\tilde{g}}_{\tilde{n}\tilde{m}}\left(\tilde{q},\tilde{t}\right)=\frac{\pi^{2}}{2}\;{\tilde{g}}_{\tilde{n}\tilde{m}}\left(\tilde{q},\tau^{*}\right)\;\;f\left(2e^{-\tau^{*}}\cos\left(\pi \tilde{n}\right)\cos\left(\pi \tilde{m}\right)\;,\frac{2}{\pi^{2}}{\tilde{q}}^{2}\right)$$

$$\mathrm{with}\;f\left(u,\lambda \right)=1-u\lambda \int_{0}^{1}dx\;x^{\lambda }e^{u\lambda (x-1)}=1+e^{-u\lambda }{\left(-u\lambda \right)}^{-\lambda }\;\gamma \left(1+\lambda ,-u\lambda \right)$$

where γ(1+λ,u) is the lower incomplete Gamma function. The other integrals have to be computed numerically.

It is possible to determine the limiting behavior of $\hat{\underline{\underline{\Lambda }}}\left(q\right)$ in certain cases: In the limit q→∞, the time integral in Equation (4) is dominated by small times $\tilde{t}$. We can then use the scaling form Equation (9) to evaluate $\underline{\underline{G}}\left(q\right)$, giving $G_{\alpha \beta }\left(q\right)=\frac{1}{D_{c}}\delta_{\alpha \beta }\frac{4f_{\alpha }N}{{\left(qR_{g}\right)}^{2}}C$ with $C=\frac{2}{\pi^{2}}\int_{0}^{\infty }xF\left(x\right)dx=3.587$. The static single chain structure factor in this limit is given by $g_{\alpha \beta }\left(q\right)=\delta_{\alpha \beta }\frac{2f_{\alpha }N}{{\left(qR_{g}\right)}^{2}}$. Putting everything together, we obtain

(13) $${\hat{\Lambda }}_{\alpha \beta }\left(q\right)\overset{q\to \infty }{⟶}\delta_{\alpha \beta }\frac{D_{c}}{k_{B}T}f_{\alpha }\cdot 0.279.$$

In the limit q→0, we specifically examine the total mobility ${\hat{\Lambda }}_{\mathrm{total}}\left(q\right)=\sum_{\alpha \beta }{\hat{\Lambda }}_{\alpha \beta }\left(q\right)$, which corresponds to the mobility function for homopolymers. The relevant contribution to the time integral entering ${\hat{\Lambda }}_{\mathrm{total}}\left(q\right)$ stems from late times, thus we can replace $\underline{\underline{g}}\left(q,t\right)$ by the asymptotic expression Equation (8), resulting in $G_{\mathrm{total}}\left(q\right)\to \frac{1}{D_{c}}\left(1-\frac{{\left(qR_{g}\right)}^{2}}{3}\right)$. Furthermore, we have $g_{\mathrm{total}}\left(q,0\right)\to N\left(1-\frac{{\left(qR_{g}\right)}^{2}}{3}\right)$ at q→0. Together, we obtain

(14) $${\hat{\Lambda }}_{\mathrm{total}}\left(q\right)\overset{q\to 0}{⟶}\frac{D_{c}}{k_{B}T}\left(1-\frac{{\left(qR_{g}\right)}^{2}}{3}\right)$$

which essentially reflects the diffusion of the whole chain. Unfortunately, a similarly simple expression for the asymptotic behavior of the individual components ${\hat{\Lambda }}_{\alpha \beta }$ for block copolymers is not available, since they also include contributions from the internal modes, which relax on time scales of order $\tau_{R}$.

**Figure 1:**
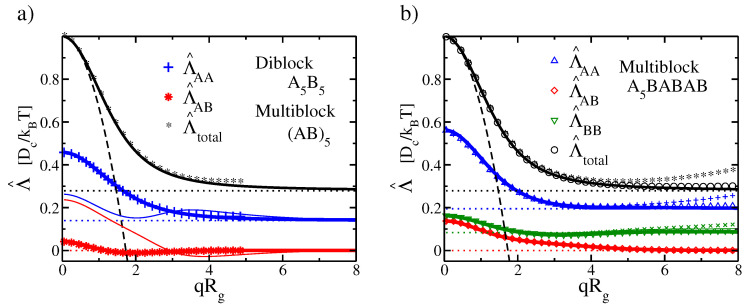
Mobility functions ${\hat{\underline{\underline{\Lambda }}}}_{\alpha \beta }\left(q\right)$ for (**a**) symmetric AB diblock copolymers and (**b**) asymmetric multiblock copolymers with sequence A${}_{5m}$B${}_{m}$A${}_{m}$B${}_{m}$A${}_{m}$B${}_{m}$. Thick solid lines show the theoretical results from Equation (4) with Equation (6). Symbols show simulation results from particle-based simulations of chains with length N=40 (plus and stars) and N=100 (circles, triangles and diamond). Dotted lines show the limiting behavior at q→∞ according to Equation (13), dashed line shows limiting behavior Equation (14) of the total mobility function at q→0. For comparison thin lines in (**a**) also show mobility functions for a symmetric multiblock with sequence (A${}_{m}$B${}_{m}$)${}_{5}$).

Figure 1 shows examples of mobility functions for three different types of linear multiblock copolymers containing two monomer species A and B. Additionally shown with dotted lines is the expected asymptotic behavior at q→∞ (Equation (13)), and with black dashed lines, the expected asymptotic behavior of ${\hat{\Lambda }}_{\mathrm{total}}$, at q→0 (Equation (14)).

Figure 1a focusses on sequences that are symmetric with respect to exchanging A and B. The thick lines show the mobility function for symmetric diblock copolymers, the thin line the corresponding results for multiblock copolymers with sequence (A${}_{m}$B${}_{m}$)${}_{5}$. In the case of diblocks, we have also carried out particle-based simulations of discrete Gaussian chains of length N=40 (symbols) for comparison. The data are in good agreement with the theory. The behavior of the dynamic mobilities of diblock and multiblock copolymers is qualitatively quite different: In diblock copolymers, the blocks move largely independent from each other: The mobility component ${\hat{\Lambda }}_{AB}$ is close to zero in the whole range of *q*. In contrast, in multiblock copolymers, the motion of blocks is highly cooperative at small *q* and they start to decouple only at $qR_{g}>1$. At q→∞, they move independent from each other as expected, i.e., ${\hat{\Lambda }}_{AB}\approx 0$.

Figure 1b shows the mobility function for a more complicated asymmetric multiblock copolymer with sequence A${}_{5m}$B${}_{m}$A${}_{m}$B${}_{m}$A${}_{m}$B${}_{m}$. It has a basic diblock structure, but one of the two blocks carries itself a periodic multiblock sequence. The mobility function combines features of the symmetric diblock and multiblock copolymer mobilities shown in Figure 1a. The behavior of the A component resembles that in regular diblock copolymers. The B component tends to move cooperatively with the A component at small *q*. The joint mobility ${\hat{\Lambda }}_{AB}$ is nonzero over a range of *q* which is even wider than in the case of pure periodic multiblock copolymers. The theoretical curves are again compared with simulation data for chains of length N=40 (plus and stars) and N=100 (circles, triangles, diamonds). The agreement is excellent at small *q*. For large *q* the simulation data for shorter chains start to deviate from the theory. This effect decreases with increasing chain length.

## 3. Application to Two-Length Scale Block Copolymers

To illustrate our DDFT approach, we will now use it to study the ordering kinetics of A${}_{5m}$B${}_{m}$A${}_{m}$B${}_{m}$A${}_{m}$B${}_{m}$block copolymer melts. They belong to a class of polymers with a two-length-scale molecular architecture, which have attracted interest as promising candidates for responsive materials [48]. In particular, linear copolymers consisting of one long uniform block and one periodic multiblock have been studied in some detail, mostly by ten Brinke and coworkers [48,49,53,54,55]. At sufficiently high χN, they form hierarchical patterns with small structures embedded in larger ones. These two length scales should be associated with two time scales, resulting in a complex ordering kinetics.

**Figure 2:**
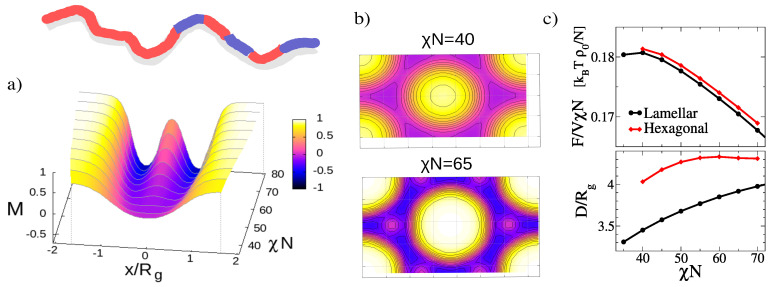
Stable and metastable ordered phases in melts of multiblock copolymers with sequence A${}_{5m}$B${}_{m}$A${}_{m}$B${}_{m}$A${}_{m}$B${}_{m}$. (**a**) Order parameter profiles for stable lamellar structures as a function of χN. A double lamellar structure emerges for χN>42.5. (**b**) Order parameter maps for selected metastable hexagonal structures for χN=40 and χN=65. The color map is the same as in panel (**a**). (**c**) Free energy per volume (**top**) and periodic distance (**bottom**) in lamellar and hexagonal phases as a function of χN. In hexagonal phases, *D* denotes distance between cylinders

To set the stage, we have determined the equilibrium structures of A${}_{5m}$B${}_{m}$A${}_{m}$B${}_{m}$A${}_{m}$B${}_{m}$copolymer melts using SCF theory for χN∈[35:80]. The results of the SCF analysis are summarized in Figure 2. The equilibrium structure is basically lamellar, but with a lamellar-in-lamellar structure emerging at χN≥42.5, characterized by an internal substructure inside the B domains. The corresponding order parameter profiles are shown in Figure 2a. Here, the order parameter is defined as $M=\left(\phi_{A}-\phi_{B}\right)/\left(\phi_{A}+\phi_{B}\right)$. The lamellar phase competes with a hexagonal phase, which also features substructures at higher χN. Examples of order parameter maps of hexagonal structures corresponding to local free energy minima are shown in Figure 2b. The SCF free energy of the lamellar phase is always slightly smaller than that of the hexagonal states, see Figure 2c. Along with the minimum free energy per volume, Figure 2c (lower panel) also shows the periodic distance/lattice parameter of the minimum structure.

Next we study the dynamic ordering process in this system using DDFT calculations with the mobility function calculated in the previous section (Figure 1b). The calculations were carried out in two dimensions in periodic boxes of side length $17.2R_{g}\times 15R_{g}$, using 86×75 grid points. These dimensions were chosen such that, for every value of χN studied here, at least one side length was roughly commensurate with the equilibrium lamellar distance and the lattice constant of the competing hexagonal pattern. The contour of the polymers was discretized with 100 ”segments”. The time step was chosen $\Delta t=\left(0.2-1.0\right)\times 10^{-4}t_{0}$ depending on the system, where the time unit is $t_{0}=R_{g}^{2}/D_{c}$. We found that the results do not depend on the precise value of the time step, as long as the simulations were stable. If the time step was too large, the numerical procedure to determine the thermodynamic forces (see Appendix A) failed, and we then reduced the time step. In most systems, $\Delta t=0.5\times 10^{-4}t_{0}$ was sufficient, but we had to set $\Delta t=0.2\times 10^{-4}t_{0}$ in the most strongly interacting systems with χN=70. For numerical reasons, we impose a frequency cutoff $\omega_{c}$, i.e., ${\left(qR_{g}\right)}^{2}{\hat{\Lambda }}_{\alpha \beta }$ may not exceed a cutoff value $\omega_{c}$. This is necessary because ${\left(qR_{g}\right)}^{2}{\hat{\Lambda }}_{\alpha \beta }$ in Equation (3) diverges at large *q* for α=β. The frequency cutoff slows down local ordering processes on very short time scales. Here, we use $\omega_{c}=5/t_{0}$. We also did shorter test runs on smaller systems with $\omega_{c}=1.5/t_{0}$ (the value found to be sufficient in our earlier work on diblock copolymers [47]) and found that the results do not change qualitatively. The initial configuration is a homogeneous melt, to which a small noise is added in order to initiate the ordering process.

**Figure 3:**
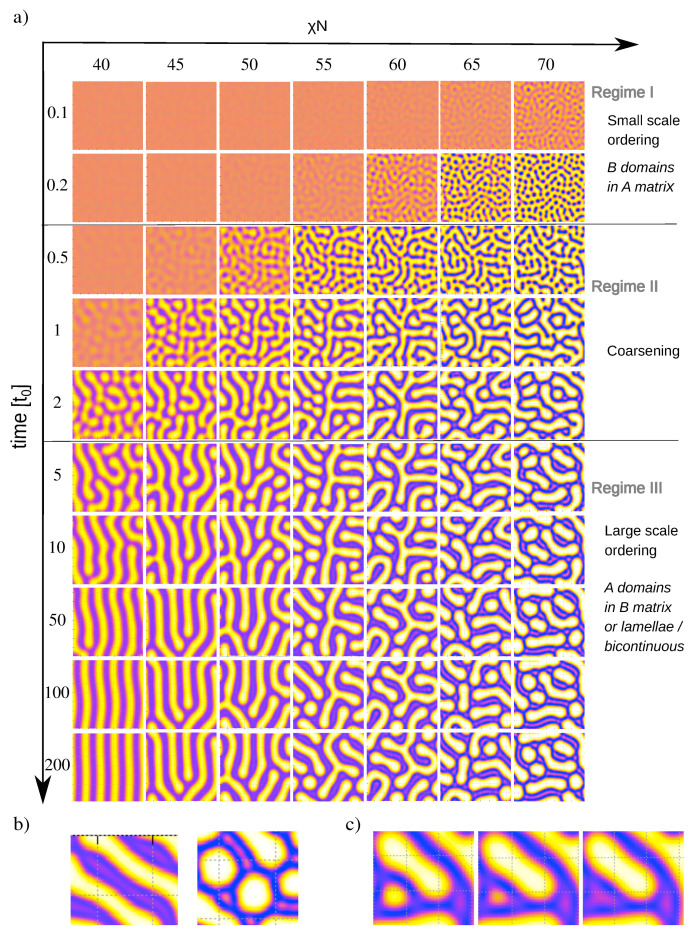
(**a**) Examples of ordering kinetics in A${}_{5m}$B${}_{m}$A${}_{m}$B${}_{m}$A${}_{m}$B${}_{m}$diblock copolymer melts for different χN and identical disordered initial configuration. The color map is the same as in Figure 2. (**b**) Details of final structures ($t=200t_{0}$) at χN=55 (**left**) and χN=70 (**right**) showing substructures in the B-domains. (**c**) Detail of a configuration where a substructure emerged by dissolution of an A domain. Parameters are χN=60 and $t/t_{0}=2.0,2.4,2.5$.

Figure 3a shows snapshots of melts during the ordering process for different χN, starting from the same initial configuration. The ordering kinetics clearly reflects the two scale character of the block copolymer. At early times $t<t_{0}$ (regime I), the local ordering is governed by the small characteristic length scale. Around $t∼t_{0}$, the structures start to coarsen (regime II), until the second characteristic length scale is reached around $t∼\left(2-5\right)t_{0}$ (regime III). The actual kinetic ordering pathway is governed by the interplay of these time-dependent ordering scales with yet another time scale, the time required for A-B segregation, which decreases with increasing incompatibility parameter χN. As a result, the ordering process depends on χN.

At low χN (e.g., χN=40), the full segregation takes place in regime III. A defective lamellar phase forms, which subsequently orders by merging of A and/or B domains, resulting in an ordered lamellar phase. At intermediate χN, the segregation starts in regime II and continues in regime III. Thus the system initially orders on small scales, then coarsens by merging of B domains, but merging of A domains is also possible. The final structure is again lamellar.

Finally, at high χN (χN>60), the system segregates already in the time regime I. It initially orders into small circular *B* domains, which then merge to form elongated connected structures. Then coarsening sets in, which is first mediated by rearrangement and further merging of B domains, and later by a thickening of B domains associated with substructure formation inside them. In most cases, A-rich substructures emerge spontaneously inside B domains. Sometimes, we also observe that a larger A island dissolves into a substructure (see Figure 3c). Once regime III is reached, the topology of the structures inverts from B domains in an A matrix (in regime I) to A domains in a B matrix. At late times, the A domains straighten out, but the basic topology of the structure no longer changes. The final structure is characterized by A domains with defined thickness but variable length, ranging from circular to elongated. Thus these final structures combine elements of the equilibrium lamellar phase and the metastable hexagonal phase. They are kinetically arrested and the topologies do no longer change. To model their further relaxation, one would have to add stochastic thermal noise to the DDFT equations, following the lines outlined in Ref. [47] (see next section). We should however note that thermal fluctuation amplitudes in copolymer melts are small [33], therefore we expect similar long-lived structures to appear in real systems as well.

## 4. Conclusions and Outlook

To summarize, in this work, we have proposed a DDFT model for studying kinetic processes of linear (multi)block copolymer melts in the Rouse regime. The model builds on earlier work [47], where we have showed how to construct such models systematically from fine-grained particle-based simulations. Here we use the same basic approach, but calculate the central input quantity— the mobility matrix—semi-analytically from the theory of Rouse dynamics. One key ingredient is an accurate approximate expression for the single chain dynamic structure factor of Rouse chains, which can be applied at all times and over a large wave vector range.

DDFT models make more approximations and are less versatile than self-consistent Brownian dynamics methods [22,23], which can also be used to study polymer systems far from equilibrium where the use of SCF free energy functional is no longer justified [26]. On the other hand, they have the advantage that they establish a natural connection to other dynamic continuum theories such as Cahn–Hilliard theories. Furthermore, they require relatively modest computational effort. For example, the calculations presented here (Figure 3) were run using own serial code on an Intel Core i7-6700 CPU processor. The CPU time per time step varied between 0.1 and 0.4 seconds, depending on the number of iterations required to determine the thermodynamic driving force self-consistently. In total, the cost for simulating one Rouse time $\tau_{R}=2/\pi^{2}t_{0}$ in our system of size 260 $R_{g}^{2}$ was roughly 12 CPU minutes for χN≤65, and 30 CPU minutes for χN=70.

To illustrate our DDFT approach, we have studied the ordering kinetics in a melt of two-length-scale copolymers. The kinetic competition on different length and time scales leads to an intricate interplay of ordering processes and results in final structures that not necessarily correspond to a true free energy minimum. Specifically, we have studied situations where an initially disordered state was instantaneously quenched into an ordered region. In that case, the final structures strongly depend on initial small fluctuations and are hard to control. A better controlled ordering process might be achieved by using a slower, well-defined and tunable quenching protocol. Experimentally, it is found that well-ordered two-scale lamellar structures can be created by quenching the samples very slowly [55]. This is consistent with our calculations where ordered structures are found to form when quenching into regions with lower χN. We are not aware of published work on non-equilibrium morphologies that can be obtained if samples are quenched more rapidly. It would be interesting to compare them with our numerical calculations. We expect that it may be possible to stabilize novel structures when quenching with specially designed quenching protocols, possibly combined with periodic re-heating. This could also be studied DDFT simulations and will be an interesting direction for future work.

In the present work, we have employed a deterministic DDFT model that ignores thermal noise. Small thermal fluctuations can be included in a straightforward manner by adding a stochastic current to the DDFT equation, i.e., replacing Eqaution (1) with

(15) $$\partial_{t}\underline{\rho }\left(r,t\right)=\nabla_{r}\left(\int dr^{\prime }\underline{\underline{\Lambda }}\left(r,r^{\prime }\right)\nabla_{r^{\prime }}\underline{\mu }\left(r^{\prime },t\right)+\underline{j}\left(r\right)\right)$$

where the components of $\underline{j}\left(r,t\right)$ are Gaussian random variables with zero mean ($\langle j_{I\alpha }\left(r,t\right)\rangle =0$) and correlations according to the fluctuation-dissipation theorem:

(16) $$\langle j_{{}_{I}\alpha }\left(r,t\right)j_{{}_{J}\beta }\left(r^{\prime },t^{\prime }\right)\rangle =2k_{B}T\delta \left(t-t^{\prime }\right)\;\Lambda_{\alpha \beta }\left(r,r^{\prime }\right)\;\delta_{{}_{IJ}}$$

(here α,β are monomer types and I,J are cartesian coordinates). Equation (15) implicitly assumes that the SCF free energy functional (from which $\underline{\mu }$ is derived) can be interpreted in the sense of a free energy landscape, which may be questionable if fluctuations are large. The relative amplitude of thermal noise is given by the inverse Ginzburg parameter [11,34] $C^{-1}=k_{B}T\;N/\rho_{0}R_{g}^{3}\propto 1/\rho_{0}\sqrt{N}$. In dense systems of polymers with high molecular weight, $C^{-1}$ is small. Thus fluctuations are small and can be neglected in many cases, except when studying very soft modes (e.g., interfacial fluctuations) and/or dynamical pathways that involve the crossing of free energy barriers.

Our illustrative DDFT calculations were carried out in two-dimensions, i.e., we have imposed uniformity in the third dimension. This was motivated by the fact that the relevant competing SCF structures of our system are one- or two-dimensional. However, in reality, the initial structures will fluctuate in all three dimensions, e.g., one will find be three-dimensional, e.g., small-scale spheres instead of small-scale cylinders. This will have to be elucidated by full three dimensional calculations.

So far, the theory is restricted to linear multiblock copolymers, and we have assumed that monomers are structurally similar, i.e., they have the same flexibility and the same monomer friction. One goal of future work will be to develop similar semi-analytic approaches for other polymer architectures, for kinetically asymmetric copolymers, or (approximately) for polymers beyond the Rouse regime.

## Abbreviations

The following abbreviations are used in this manuscript:

BCP	Block copolymer
DDFT	Dynamic density functional theory
SCF	Self consistent field

## Appendix A. SCF Equations

In the following, we briefly summarize the main SCF equations for our system. More detailed discussions of the SCF theory can be found, e.g., in Refs. [9,10,11,12]. In the SCF approximation, the free energy functional $F\left[\{\phi_{\alpha }\left(r\right)\}\right]$ is expressed as

(A1) $$\begin{array}{c} \frac{F}{k_{B}T}=\frac{\rho_{0}}{N}\left\{\int dr\left[\chi N\;\phi_{A}\left(r\right)\phi_{B}\left(r\right)+\kappa N{\left(\phi_{A}\left(r\right)+\phi_{B}\left(r\right)-1\right)}^{2}\right]-\int dr\;\underline{\phi }\left(r\right)\cdot \underline{\omega }\left(r\right)-V\ln Q\right\} \end{array}$$

where $\underline{\phi }=\left\{\phi_{\alpha }\right\}$ denotes the vector of normalized density field of monomers of type α, $\underline{\omega }=\left\{\omega_{\alpha }\right\}$ the corresponding vector of conjugate fields, and *Q* the single chain partition function in the external fields $\underline{\omega }$. The conjugate fields are defined implicitly by the requirement

(A2) $$\underline{\phi }\left(r\right)=\frac{V}{Q}\int_{0}^{1}d\tilde{n}\;\underline{\chi }\left(\tilde{n}\right)\;q_{f}\left(r,\tilde{n}\right)\;q_{b}\left(r,1-\tilde{n}\right)$$

where the chain propagators $q_{f}\left(r,s\right)$ and $q_{b}\left(r,s\right)$ are obtained from solving the differential equations

(A3) $$\begin{array}{ccc} \frac{\partial q_{f}\left(r,\tilde{n}\right)}{\partial \tilde{n}} & = & R_{g}^{2}\nabla^{2}q_{f}\left(r,\tilde{n}\right)-\underline{\omega }\left(r\right)\cdot \underline{\chi }\left(\tilde{n}\right)\;q_{f}\left(r,\tilde{n}\right) \end{array}$$

(A4) $$\begin{array}{ccc} \frac{\partial q_{b}\left(r,\tilde{n}\right)}{\partial \tilde{n}} & = & R_{g}^{2}\nabla^{2}q_{b}\left(r,\tilde{n}\right)-\underline{\omega }\left(r\right)\cdot \underline{\chi }\left(1-\tilde{n}\right)\;q_{b}\left(r,\tilde{n}\right) \end{array}$$

with initial condition $q_{f,b}\left(r,0\right)=1$, and the single chain partition function *Q* is given by

(A5) $$Q=\frac{1}{V}\int dr\;q_{f}\left(r,1\right)=\frac{1}{V}\int dr\;q_{b}\left(r,1\right)$$

The SCF equilibrium state is obtained by minimizing $F\left[\{\phi_{\alpha }\left(r\right)\}\right]$, leading to a second set of equations for $\underline{\omega }\left(r\right)$:

(A6) $$\omega_{A}^{SCF}\left(r\right)=\chi N\phi_{B}+2\kappa N\left(\phi_{A}+\phi_{B}-1\right),\;\omega_{B}^{SCF}\left(r\right)=\chi N\phi_{A}+2\kappa N\left(\phi_{A}+\phi_{B}-1\right)$$

In DDFT calculations, these conditions are replaced by equations for the thermodynamic fields that drive the system towards equilibrium,

(A7) $${\hat{\underline{\mu }}}_{\alpha }\left(r\right)=\left({\underline{\omega }}^{SCF}\left(r\right)-\underline{\omega }\left(r\right)\right)$$

In SCF calculations, one must solve self-consistently the set of Equations (A2) and (A6). In DDFT calculations, one must solve Equation (A2) for the conjugated fields for given density fields $\phi_{\alpha }\left(r,t\right)$ in every time step. Both tasks require iterative methods. In the present work, we use Anderson mixing [56] and a pseudospectral method for solving the propagator Equations (A3) and (A4) [33]. In the DDFT calculations, we require an accuracy of $\max_{\alpha r}\left|\Delta \phi_{\alpha }\left(r\right)\right|<0.001$ in every time step, where $\Delta \underline{\phi }$ is the difference between the target density profile and the profile obtained from Equation (A2) with the given fields $\underline{\omega }$. In the SCF calculations, we require $\max_{\alpha r}\left|\Delta \omega_{\alpha }\left(r\right)\right|<10^{-6}$ for the difference $\Delta \underline{\omega }$ between the initial value of $\underline{\omega }$ in an iteration step and the final value computed from Equations (A2) and (A6).

## Appendix B. Single Chain Dynamic Structure Factor

### Appendix B.1. Late Time Behavior

We consider the dynamic structure factor of single non-interacting Gaussian chains in the Rouse regime. Since all variables are Gaussian distributed, Equation (5) can be rewritten in the form

(A8) $$g_{\alpha \beta }\left(q,t\right)=\frac{1}{N}\;\int \int_{0}^{1}\;\;\;dn\;dm\;\chi_{\alpha }\left(\frac{n}{N}\right)\;\chi_{\beta }\left(\frac{m}{N}\right)\;\exp\left(-\frac{1}{6}q^{2}\langle {\left(R_{n}\left(t\right)-R_{m}\left(0\right)\right)}^{2}\rangle \right)$$

The trajectories $R_{n}\left(t\right)$ can be split up into the center of mass motion $R_{c}\left(t\right)$ and the relative motion $r_{n}\left(t\right)$,

(A9) $$R_{n}\left(t\right)=R_{c}\left(t\right)+r_{n}\left(t\right)\;\mathrm{with}\;R_{c}\left(t\right)=\frac{1}{N}\int_{0}^{N}\;\;\;dn\;R_{n}\left(t\right)$$

In the limit t→∞, the internal coordinates $r_{n}\left(t\right)$ and $r_{m}\left(0\right)$ are uncorrelated with each other and with $R_{0}$, i.e.,

$$\langle {\left(R_{n}\left(t\right)-R_{m}\left(0\right)\right)}^{2}\rangle \approx \langle {\left(R_{0}\left(t\right)-R_{0}\left(0\right)\right)}^{2}\rangle +\langle r_{n}^{2}\rangle +\langle r_{m}^{2}\rangle$$

The first term describes the center of mass diffusion of the chain and obeys $\langle {\left(R_{0}\left(t\right)-R_{0}\left(0\right)\right)}^{2}\rangle =6D_{c}t$. The second term describes the monomer fluctuations about the center of mass. It can be calculated using the relation $\langle {\left(R_{n}-R_{m}\right)}^{2}\rangle =6\left|n-m\right|R_{g}^{2}/N$, which is valid for Gaussian chains. After some algebra, one obtains $\langle r_{n}^{2}\rangle =6R_{g}^{2}\;\left({\left(\frac{n}{N}\right)}^{2}-\frac{n}{N}+\frac{1}{3}\right)$. Thus the dynamic structure factor takes the asymptotic form $\underline{\underline{g}}\left(q,t\right)\approx N\;e^{-q^{2}D_{c}t}\;\underline{I}\left(qR_{g}\right)⊗\underline{I}\left(qR_{g}\right)$ with

(A10) $$\;\underline{I}\left(\tilde{q}\right)=\langle \int_{0}^{N}\;\;\;dn\;\underline{\chi }\left(\frac{n}{N}\right)\;e^{iq\cdot r_{n}}\rangle =\int_{0}^{1}\;\;\;d\tilde{n}\;\underline{\chi }\left(\tilde{n}\right)\;e^{-{\tilde{q}}^{2}\;\left({\tilde{n}}^{2}-\tilde{n}+1/3\right)}$$

which corresponds to Equation (8).

### Appendix B.2. Derivation of an Approximation for $\underline{\underline{g}}\left(q,t\right)$

At finite *t*, the dynamic correlations due to internal modes can no longer be ignored. The exact solution for g(q,t) then involves an infinite sum over all Rouse modes *p*. For homopolymers, the resulting expression for homopolymers is derived, e.g., in Ref. [51] (Appendix 4. III). It can easily be generalized to linear multiblock copolymers, giving

(A11) $$\underline{\underline{g}}\left(q,t\right)=e^{-q^{2}D_{c}t}\;N\;\;\int \int_{0}^{1}\;\;\;d\tilde{n}\;d\tilde{m}\;\underline{\chi }\left(\tilde{n}\right)⊗\underline{\chi }\left(\tilde{m}\right)\;e^{-{\left(qR_{g}\right)}^{2}\left(|\tilde{n}-\tilde{m}|+H\left(\tilde{n}-\tilde{m},t/\tau_{R}\right)+H\left(\tilde{n}+\tilde{m},t/\tau_{R}\right)\right)}$$

with

(A12) $$H\left(x,\tau \right):=\frac{2}{\pi^{2}}\sum_{p=1}^{\infty }\frac{1}{p^{2}}\;\cos\left(p\pi x\right)\;\left(1-e^{-\tau p^{2}}\right)$$

Our goal is to approximate the function H(x,τ). We first note that it can be evaluated exactly in the limit τ→∞, giving

(A13) $$H\left(x,\infty \right)=\frac{2}{\pi^{2}}\sum_{p=1}^{\infty }\frac{1}{p^{2}}\cos\left(p\pi x\right)=\left[\frac{1}{2}{\left(x-1\right)}^{2}-\frac{1}{6}\right]\;\mathrm{mod}\;2=:H_{\infty }\left(x\right)$$

To prove this relation, one simply calculates the coefficients of the Fourier series of $H_{\infty }\left(x\right)$. The leading correction term at τ≫1 is the term proportional to $e^{-\tau }$, hence we obtain

(A14) $$H\left(x,\tau \right)\approx H_{\infty }\left(x\right)-\frac{2}{\pi^{2}}e^{-\tau }\;\cos\left(\pi x\right)\;\mathrm{at}\;\tau \gg 1$$

Likewise, we can evaluate the behavior of H(x,τ) exactly in the limit of τ→0 and small |x|≪1. In that limit, one can replace the sum over *p* by an integral, giving

(A15) $$H\left(x,\tau \right)\approx \frac{2}{\pi^{2}}\int_{0}^{\infty }\;\;\;dp\;\frac{1}{p^{2}}\;\cos\left(p\pi x\right)\left(1-e^{-\tau p^{2}}\right)=\sqrt{\tau }\;\Phi \left(x/\sqrt{\tau }\right)$$

(A16) $$\mathrm{with}\;\Phi \left(y\right)=\frac{2}{\pi^{2}}\;\left(e^{-\frac{1}{4}{\left(\pi y\right)}^{2}}\sqrt{\pi }-\frac{\pi^{2}}{2}|y|\;(1-\mathrm{Erf}(\pi |y|/2))\right)$$

where Erf is the error function. Next we seek to approximate H(x,τ) over the whole range x∈[−2:2] for finite small τ≪1. To this end, we exploit the relation H(x,τ)=H(|x|,τ)=H(2−|x|,τ) and make the Ansatz $H\left(x,\tau \right)\approx H_{c}\left(x,\tau \right)+C\left(\tau \right)$ with

(A17) $$H_{c}\left(x,\tau \right)=\sqrt{\tau }\;\left(\Phi \left(\frac{\left|x\right|}{\sqrt{\tau }}\right)+\Phi \left(\frac{2-|x|}{\sqrt{\tau }}\right)\right)$$

where we choose the offset C(τ) such that the approximation is best at x=1, i.e., $C\left(\tau \right)=H\left(1,\tau \right)-H_{c}\left(1,\tau \right)$. Thus we need to find a good approximation for H(1,τ) at finite small τ. We know H(1,0)=0 and

(A18) $$\partial_{\tau }H\left(1,\tau \right)=\frac{2}{\pi^{2}}\sum_{p=1}^{\infty }{\left(-1\right)}^{p}\;\;e^{-\tau p^{2}}=\frac{1}{\pi^{2}}\left(\theta_{4}\left(e^{-\tau }\right)-1\right)\overset{\tau \to 0}{⟶}-\frac{1}{\pi^{2}}$$

where $\theta_{4}\left(y\right)$ is the Theta “constant”. Numerically, it turns out that $\theta_{4}\left(e^{-\tau }\right)$ closely follows

(A19) $$\theta_{4}\left(e^{-\tau }\right)\approx 2\sqrt{\frac{\pi }{t}}e^{-\pi^{2}/4t}=\pi^{2}\partial_{\tau }H_{c}\left(1,\tau \right)$$

over a fairly large range of τ>0. The deviation from the exact value of $\theta_{4}\left(e^{-\tau }\right)$ scales roughly as $e^{-20/t}$. Thus we can approximate $\partial_{\tau }H\left(1,\tau \right)\approx -1/\pi^{2}+\partial_{\tau }H_{c}\left(1,\tau \right)$ and hence $H\left(1,\tau \right)\approx -\frac{1}{\pi^{2}}\;\tau +H_{c}\left(1,\tau \right)$. Inserting this into our Ansatz above, we obtain

(A20) $$H\left(x,\tau \right)\approx H_{c}\left(x,\tau \right)-\tau /\pi^{2}\;at\;\tau \ll 1$$

Starting from the two limiting expressions, Equations (A20) and (A14), we now make the overall Ansatz

(A21) $$H_{\mathrm{appr}}\left(x,\tau \right)=\left\{\begin{array}{cc} H_{c}\left(x,\tau \right)-\tau /\pi^{2} & :\tau <\tau^{*}\\ H_{\infty }\left(x\right)-\frac{2}{\pi^{2}}e^{-\tau }\cos\left(\pi x\right) & :\tau >\tau^{*} \end{array}\right.$$

We choose the crossover value $\tau^{*}=1.7$ such that it minimizes the maximum absolute value of the deviation $\Delta_{H}\left(x,\tau \right)=H_{\mathrm{appr}}\left(x,\tau \right)-H\left(x,\tau \right)$ between the approximate expression (A21) and the numerical evaluation of the full expression, Equation (A12). With this approach, we can reach $\max(|\Delta_{H}\left(x,\tau \right)\left|\right)\leq 10^{-4}$ over the whole range of *x* and τ. Figure A1 shows H(x,τ) and the $\Delta_{H}\left(x,\tau \right)$ as a function of *x* and τ.

Inserting $H_{\mathrm{appr}}\left(x,\tau \right)$ into Equation (A11) and performing some further simple transformations, we finally obtain Equation (6) in the main text. From $\max\left(\Delta_{H}\right)\leq 10^{-4}$, we can estimate that the relative error of $g_{\alpha \beta }\left(q,t\right)$ is less than $2{\left(qR_{g}\right)}^{2}\times 10^{-4}$ over the whole range of q and *t*.
